# Individual and collective contribution of antenatal psychosocial distress conditions and preterm birth in Pakistani women

**DOI:** 10.1371/journal.pone.0282582

**Published:** 2023-03-30

**Authors:** Sharifa Lalani, Shahirose Sadrudin Premji, Kiran Shaikh, Salima Sulaiman, Ilona S. Yim, Ntonghanwah Forcheh, Neelofur Babar, Sidrah Nausheen, Nicole Letourneau, the Maternal-infant Global Health Team (MiGHT) Collaborators in Research

**Affiliations:** 1 School of Nursing and Midwifery, The Aga Khan University, Karachi, Pakistan; 2 Faculty of Health Sciences, School of Nursing, Queen’s University, Kingston, Ontario, Canada; 3 Faculty of Nursing, Brock University, St. Catharines, Ontario, Canada; 4 Department of Psychological Science, University of California, Irvine, Irvine, California, United States of America; 5 Department of Obstetrics and Gynaecology, The Aga Khan Hospital for Women, Karimabad, Karachi, Pakistan; 6 Department of Obstetrics and Gynaecology, The Aga Khan University, Karachi, Karachi, Pakistan; 7 Faculty of Nursing & Cumming School of Medicine, Departments of Pediatrics, Psychiatry & Community Health Sciences, University of Calgary, Calgary, Alberta, Canada; Universita Cattolica del Sacro Cuore Sede di Roma, ITALY

## Abstract

**Background:**

We determined whether dimensions of psychosocial distress during pregnancy individually and collectively predicted preterm birth (PTB) in Pakistani women as it may be misleading to extrapolate results from literature predominantly conducted in high-income countries.

**Methods:**

This cohort study included 1603 women recruited from four Aga Khan Hospital for Women and Children in Sindh, Pakistan. The primary binary outcome of PTB (i.e., livebirth before 37 completed weeks’ gestation) was regressed on self-reported symptoms of anxiety (Pregnancy-Related Anxiety (PRA) Scale and Spielberger State-Trait Anxiety Inventory Form Y-1), depression (Edinburgh Perinatal Depression Scale (EPDS)), and covariates such as chronic stress (Perceived Stress Scale) assessed with standardized question and scales with established language equivalency (Sindhi and Urdu).

**Results:**

All 1603 births occurred between 24 and 43 completed weeks’ gestation. PRA was a stronger predictor of PTB than other types of antenatal psychosocial distress conditions. Chronic stress had no effect on the strength of association between PRA and PTB and a slight but non-significant effect on depression. A planned pregnancy significantly lowered risk of PTB among women who experienced PRA. Aggregate antenatal psychosocial distress did not improve model prediction over PRA.

**Conclusions:**

Like studies in high-income countries, PRA became a strong predictor of PTB when considering interactive effects of whether the current pregnancy was planned. Women’s resilience and abilities to make sexual and reproductive health decisions are important to integrate in future research. Findings should be generalized with caution as socio-cultural context is a likely effect modifier. We did not consider protective/strength-oriented factors, such as resilience among women.

## Introduction

Preterm birth, defined as live birth before 37 weeks of gestation [1], is a global public health issue as it is one of the leading causes of morbidity as well as preventable deaths of newborns and children less than 5 years of age [2]. The global preterm birth rate is estimated to be 10.6% or almost 15 million preterm birth births annually worldwide [3]. A systematic review and meta-analysis of Pakistani studies reported an 18.9% pooled prevalence of preterm birth [4], with rates as high as 22.8% in some rural communities [5]. There are multiple reported risk factors for preterm birth (e.g., maternal age, parity, previous preterm birth, terrible events in neighborhood) [6–11]. Of particular concern in low- and middle-income countries (LMIC) is the higher prevalence of antenatal psychosocial distress which refers to emotional suffering exhibited as depressive and/or anxiety symptoms [12]. A recent systematic review of 25 Pakistani studies (10,368 women) estimated the pooled prevalence of antenatal depression at 37% (95% CI: 30–44%) [13], which is higher than the prevalence rate among LMIC (25.3%, 95% CI: 21.4–29.6%) [14] and South Asian countries (24.3%, 95% CI: 19.03–30.47%) [15]. However, the prevalence of antenatal depression among Pakistani women varies significantly (10–75%) [16–18] with differences attributed to setting, screening instruments, and unique contextual factors [19, 20].

A recent umbrella review estimated the global risk of preterm birth to be 1.49 (95% CI: 1.32–1.68; I^2^ = 0.0%) times higher among pregnant mothers with antenatal depression [12]. Studies from high-income countries examining the relationship between antenatal depression and preterm birth have shown inconsistent findings, with a minority of the studies [21–23] finding a statistically significant association with a small effect size [24, 25]. Perceived stress during pregnancy, a proxy of chronic stress, is associated with depressive symptoms as well as preterm birth [26–29] and may moderate the relationship between antenatal depressive symptoms and preterm birth [30]. Although our pilot study of Pakistani women found that antenatal depression strongly predicted preterm birth (odds ratio (OR) 1.44), the degree to which antenatal anxiety predicts preterm birth in this population is unknown [31].

Systematic reviews and meta-analyses reveal inconsistent findings for an association between maternal anxiety during pregnancy and preterm birth [32, 33]. Maternal antenatal anxiety includes state anxiety and pregnancy-related anxiety, with the former encompassing situations or circumstances that elicit temporary or emotional anxiety [34, 35]. Pregnancy-related anxiety, a mental state in which pregnant women have pregnancy-related concerns, such as fears of delivery and health of the child [36], is not always identified as a distinct type of distress when examining the association between antenatal psychosocial distress and preterm birth [37, 38]. The available literature from high-income countries suggests that the association between pregnancy-related anxiety and preterm birth is evident in diverse income and ethnic groups [24, 39], and is a stronger determinant of preterm birth than general anxiety or worry about life events [23, 24, 40–43]. Our own systematic review and meta-analysis did not support this assertion but noted further reduction in heterogeneity when restricting the predictor variable to state anxiety (OR = 1.70, 95% CI: 1.33–2.18) and pregnancy-related anxiety (OR = 1.67, 95% CI: 1.35–2.07) [39], and explained the contradictory findings between two meta-analyses examining maternal anxiety in pregnancy and preterm birth [32, 33]. In our pilot observational cohort study mother’s concerns regarding fetal wellbeing showed a trend of predicting preterm birth [30].

The social, cultural, and environmental contexts of LMIC produce more extreme and prolonged exposure to stressors (i.e., chronic stress) [44, 45], thereby inducing greater antenatal psychosocial distress [12]. For example, Pakistani studies identified culture-specific predictors of unplanned pregnancy to be sex preference, restrictions on women’s access to health care and agency to make decisions regarding family planning, and limited sexual and reproductive health literacy [46]. Prior research of our team member revealed that women’s husbands or in-laws declined permanent methods of contraception (e.g., tubal ligation) contrary to women’s decision and even when no further children were desired [47]. Pakistani women who have less autonomy are likely to embrace traditional attitudes and values regarding pregnancy [48]. For example, women believe that breastfeeding would protect against pregnancy. Sex of the older child [46], specifically having a son, increased the likelihood of unplanned pregnancies to increase the number of breadwinning sons in the family [48]. Women’s abilities to make decisions regarding their reproductive health, a vital aspect of women’s empowerment and pregnancy-related empowerment, impacts antenatal anxiety [49, 50]. Pakistani women are likely more vulnerable to antenatal depression and anxiety due to intersecting socioeconomic factors (e.g., poverty, unemployment, lack of education, inflation), obstetric factors (e.g., multiparity, unplanned pregnancy), psychological factors (e.g., abuse perpetrated by husbands and mother-in-law), and sociocultural factors (e.g., societal/family pressure to give birth to male, worries about giving birth to female) [20, 51–53]. We, therefore, consider the socio-cultural context of women when examining the relationship between antenatal psychosocial distress and preterm birth.

Depression, state anxiety and pregnancy-related anxiety may be sequelae of conditions. For example, pregnancy-related anxiety may be a natural consequence of state anxiety as it has been found to be more prevalent among women who report high state anxiety [39, 54, 55]. Pregnancy-related anxiety, state anxiety and depression scores are significantly correlated (*r* = 0.45–0.68) [33, 56], and one in 10 women were reported to experience some form of comorbid anxiety and depression in a systematic review and meta-analysis of 66 studies involving 162,120 women across 30 countries [57]. When depression, pregnancy-related anxiety, state anxiety, or perceived stress co-occur in any combination during pregnancy, the risk of preterm birth increases [28, 58]. However, studies conducted in high-income countries fail to consider collinearity between various dimensions of psychosocial distress when examining the relationship between antenatal psychosocial distress and preterm birth, thus making it difficult to distinguish the effects of various dimensions of antenatal psychosocial distress on preterm birth [39]. A systematic review and meta-analysis of 16 studies conducted in high-income countries examining mixed exposure (i.e., two or more antenatal psychosocial distress measures of depression, anxiety, or perceived stress) reported an increase in risk of preterm birth; however, only five studies identified pregnancy-related anxiety as a distinct construct [28].

The purpose of the study was to examine (a) all three antenatal psychosocial distress conditions–depressive symptoms, state anxiety, and pregnancy-related anxiety–in the model for preterm birth, and (b) the relationship between the collective contribution of antenatal psychosocial distress conditions and preterm birth. We hypothesized pregnancy-related anxiety to be a stronger predictor of preterm birth in Pakistani pregnant women than other psychosocial distress conditions as high maternal and neonatal mortality and socio-economic and cultural barriers to access timely and effective health service would contribute to fears of delivery and health of the child [36]. We also ascertained if, in combination with chronic stress, the strength of the association between antenatal psychosocial distress condition(s) and preterm birth was increased.

## Methods

### Ethics statement

The National Bioethics Committee (NBC) Pakistan [No.4-87/NBC-286-Y2] and the Aga Khan University Ethics Review Committee [5003-SON-ERC-17], Karachi, Pakistan approved the study. Ethics approval was also secured from: Mount Royal University Human Research Ethics Board [File ID#101116], University of Calgary Conjoint Health Research Ethics Board [REB17-1148_REN5], York University Office of Research Ethics [2018–184], and Queen’s University Health Sciences & Affiliated Teaching Hospitals Research Ethics Board [NURS-566-23]. Each participant provided informed (oral or written) consent based on preference of language and literacy level. Information in the consent form was orally narrated in Urdu, the preferred language, permission documented in the consent form, and a copy of the consent form provided to participants as per procedures approved by the ethics committee.

### Study design, setting, and sample

**Fig 1:**
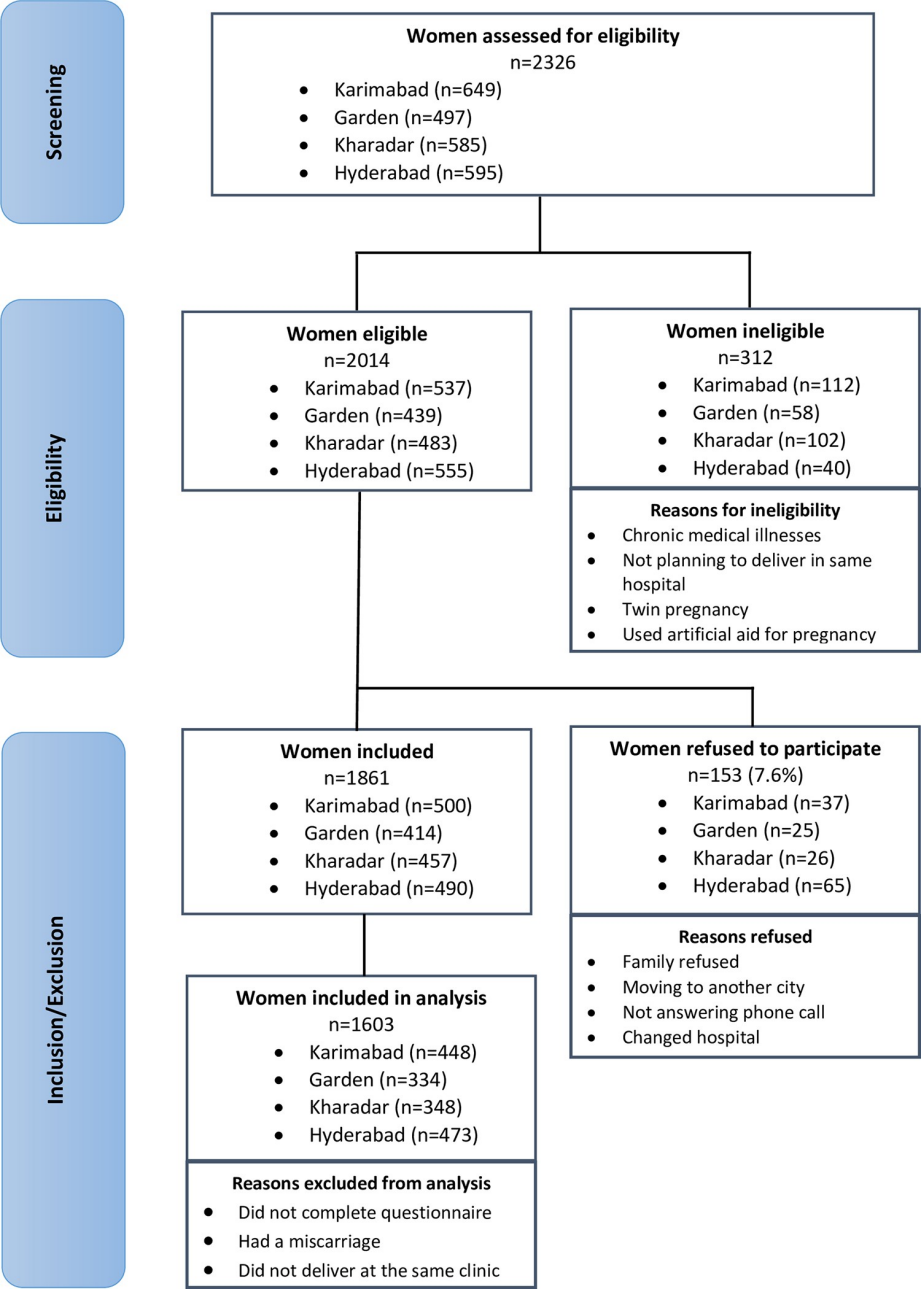


A prospective cohort study recruited 1861 healthy pregnant women aged 18–42 years (27.1 ± 4.8 years) and within 10–19 weeks’ gestation at enrolment from women seeking antenatal care at any one of four Aga Khan Hospitals for Women and Children (AKHWC) in Sindh Province, Pakistan–Karimabad, Garden, Kharadar, Hyderabad. The study started in February 2018 and the sample size was attained in February 2020. After providing informed consent, participants completed self-report questionnaires at enrolment and at an antenatal follow-up visit at 22–29 weeks’ gestation. Women were followed until birth and birth outcome data were collected during delivery for all women who returned to their respective clinics for delivery. Fig 1 shows the flow of participants through the study.

AKHWC is a university-affiliated teaching hospital that delivers 8,000 infants per year, and collectively serves women representing the ethnic (mostly Urdu-Muhajir) and socio-economic diversity (17% low-income) of Karachi [59]. Each of the four centres offers psychiatric services to which 13% (n = 245) of the women enrolled were referred at no cost to them, to manage their perinatal mental health during the duration of the study; however only 1% (n = 19) accessed the service.

We estimated the sample size using the Stata Program powerlog, based on a multivariable logistic regression model of the primary research questions thereby accounting for modelling of up to 8 predictor variables with the outcome of spontaneous preterm birth, allowing for possible correlations and unknown prevalence of pregnancy-related anxiety though anticipated to be higher than depression. A Bonferroni correction for an overall alpha level of 0.00625 was applied. Using a 2-sided test with 85% power, the required sample size to detect an OR of 1.44 based on our pilot study [31] (corrected prevalence 12.5% and 9%, respectively, to restrict to spontaneous preterm birth [60]), for a 1 standard deviation change in the predictor variable was 1404 assuming a squared multiple correlation of 0.15. To account for estimates of potential loss to follow-up and missing data, we overenrolled for a total sample of 1861. Missing data arose only for enrolled women who (a) did not deliver at the same clinic and who thus were excluded as they did not meet our inclusion criteria, or (b) had a miscarriage thus were excluded given our definition of preterm birth (≥ 22 and < 37 weeks’ gestational age).

### Inclusion and exclusion criteria

Women were enrolled if they had a singleton pregnancy at 10–19 weeks’ gestation (based on last menstrual period or ultrasound if unsure of last menstrual cycle before being pregnant), had no known pre-existing conditions that would affect their health and the health of their baby (e.g., diabetes mellitus, thyroid disorder, mental health disorders), were willing to return for assessment at 22–29 weeks’ gestation, planned to return to deliver their baby at AKHWC, and were able to speak Urdu, Sindhi, or English. Women were excluded if they were victims of terrorism (e.g., political, religious, or ideological war, violence, threats), used psychotropic medications, achieved pregnancy with the aid of artificial reproductive technologies, or were diagnosed with HIV/AIDS.

### Measures

The primary outcome of interest was preterm birth defined as live born infants ≥ 22 to < 37 completed weeks’ gestation, derived from gestational age at enrolment. Infants born 37 to 42 weeks’ gestation were categorized as term infants. Standardized questions and scales to measure predictors and covariates were assessed by team members for content and face validity. Further, they were modified as appropriate *a priori* by removing questions deemed irrelevant or culturally insensitive. Content was translated into Urdu and Sindhi, and back translated by an independent translator to English for language equivalency by comparing the translation to the original content. The questions and scales were pilot tested with the target population before administration in the study.

### Antenatal psychosocial distress predictor variables

Self-reported symptoms of antenatal psychosocial distress were determined with three instruments, all of which use 4-point Likert scales: (1) Pregnancy-Related Anxiety Scale, (10-item, range 10–40, cut-off of 22; Cronbach’s α = 0.78) which evaluated the current feelings about health during pregnancy, health of fetus/infant, labor and delivery [61]; (2) Spielberger State-Trait Anxiety Inventory Form Y-1 (20-items; range 20–80, cut-off of 50, Cronbach’s α = 0.86–0.94) which assessed temporary or emotional anxiety occurring “right now” as a result of situational circumstances [34, 35, 62]; and (3) Edinburgh Perinatal Depression Scale (10-item, range 0–30, cut-off 10, across 15 countries including some LMIC Cronbach’s α = 0.73–0.87; 3 to 12 week test-retest = 0.53–0.74) which assessed depressive feeling over the past 7 days [63–68].

### Self-reported measure of covariates

At enrollment participants provided data on potential covariates identified from evidence in the literature along with scientific and clinical judgment (i.e., in the case of a ‘trend’ toward association) [69]. A self-reported questionnaire collected information on demographic factors (e.g., age, ethnicity, income, education), behavioral factors (e.g., smoking, alcohol use, illicit drug use), pre-pregnancy characteristics (e.g., medical conditions, pre-pregnancy weight), pregnancy characteristics (e.g., pregnancy spacing, unplanned pregnancy, parity, obstetric risks), and socio-cultural factors (e.g., domestic violence, social support). All women were married; therefore, marital status was excluded for lack of variability and replaced with age at first marriage. The Perceived Stress Scale (10-item with 5-point Likert type questions; range 0–40, cut-off 20; Cronbach’s α = 0.78–0.91) available in Urdu served as a proxy measure of chronic stress over the past month [70–73].

### Statistical analysis

SPSS (version 25) was used to perform all the analyses. Multiple logistic regression was employed to answer the research question as the outcome variable, preterm birth, was a binary variable (1 = preterm, 0 = term). The sample was characterized with descriptive statistics. The predictor variables comprised the three antenatal psychosocial distress measures, namely pregnancy-related anxiety, state anxiety, and depression. A separate analysis used an aggregate score of the three measures as the sole predictor in line with the secondary research question. Each predictor was dichotomised using the recommended cut-offs (1 = anxiety/depressive symptom, 0 = no anxiety/depressive symptom). The small number of predictors (3) and the weak correlation between them (range of *r* = 0.330 to 0.481) meant that multicollinearity was not of concern. The aggregate score ranged from 0 to 3 (0 = no anxiety/depression, 1 = any one of the conditions, 2 = any two of the conditions, 3 = all 3 conditions). However, it was dichotomised as 1 if women experienced one or more of the 3 conditions and as 0 otherwise because the number of women with comorbid conditions and preterm birth was very small.

### Confounders and effect modifiers

We assessed socio-demographic and behavioral variables as potential confounders or effect modifiers for each given predictor separately (see S1 Table). Any risk factor/covariate that was found to be associated (*p* < 0.10) with both preterm birth outcome and antenatal psychosocial distress predictor variable was interpreted as a potential confounder, while any found to be associated with only one of the preterm birth or predictor variable was interpreted as potential effect modifier. For categorical risk factors, all bivariate associations were evaluated using chi-squared tests of association while quantitative covariates (e.g., age) were evaluated using odds-ratios.

### Models for preterm birth

We used three hierarchical logistic regression models to estimate crude and adjusted odd ratios for preterm birth. Each hierarchical model started with the predictor(s) as the sole variable in the model to obtain the crude effects, then adding all potential confounders between preterm birth and the predictor(s) to adjust crude effects for any confounding. In the third and final model, all potential effect modifiers together with their interaction terms with the predictor variable(s) were added to the model with confounders (if any). The forward likelihood criterion was used to retain only statistically significant effects in each level of the hierarchical model.

The first model was used to determine if each measure of psychosocial distress during pregnancy (pregnancy-related anxiety, state anxiety, depression) individually predicted preterm birth. The second model was to determine if measures of psychosocial distress during pregnancy collectively predicted preterm birth. Two approaches were used to quantify the collective effects. In the first approach, the 3 measures and their interaction terms were included in the hierarchical multiple logistic regression models. In the second approach, a composite binary measure was computed to measure the combined effect and coded as 1 if any psychosocial distress condition was present and 0 if none were present. Only significant confounders and effect modifiers retained from each of 3 models for individual psychosocial distress as predictor were evaluated in both approaches.

For pregnancy-related anxiety, state anxiety, and depression, effect modifiers were quantified and interpreted through interaction effects between preterm birth and each predictor variable. The Akaike information criterion (AIC) criterion [74] was used to compare the 3 final models. We also investigated if perceived stress was a potential effect modifier for any of the 3 antenatal psychosocial distress conditions in this analysis. Only significant effects were retained in the final model.

## Results

Our sample size comprises the 1603 (86.2%) women who returned and gave birth (53.3% boys) at their clinics (Fig 1). Their mean age at the time of enrolment was 27.1 years (SE = 0.119). Almost all women (99.9%) were married with some women (20.7%) marrying at an age of less than 20 years. Although many women (60.7%) did not choose their husbands, majority of the women (98.1%) indicated they had a voice in the decision making (i.e., gave consent for marriage). All ethnic groups in Pakistan were represented, with Urdu-Mahajir (31.1%), Sindhi (23.6%) and Memon (12.5%) being the dominant ethnicities. Majority of the women (89.1%) were homemakers. Economic vulnerability was evident with women self-reporting low total family income (17%) and low socio-economic status (7.9%). However, about a third of the women (33.7%) had postgraduate education and a further 38.7% had college or university degrees. Risky health behaviors prior to or during pregnancy that were prominent among women included drug (13.5%) and substance use (14.7%); none of the women reported alcohol use. Many women (40%) scored high (i.e., cut-off 20) on the Perceived Stress Scale which was a proxy of chronic stress. Women were supported by family (95.1%), friends (84.9%), and/or had other sources of support (99.4%). The prevalence of state anxiety, pregnancy-related anxiety, and depressive symptoms were 2.7% (n = 44), 7.3% (n = 117), and 12.9% (n = 206), respectively. Using the raw scores, the lowest correlation was between pregnancy-related anxiety and state anxiety (*r* = 0.330, *p* < 0.001). The correlation coefficients ranged from 0.330 to 0.481. Table 1 provides further details about the demographic, behavioral factors, pre-pregnancy and pregnancy characteristics, and socio-cultural context of our cohort, and prevalence of preterm birth.

**Table 1:** Characteristics of cohort and prevalence of preterm birth.

Covariates and predictors		n (%)	Preterm birth prevalence (%)
		1603 (100%)	13.0
Age at enrolment			
	Under 20 years	86 (5.4)	10.5
	20–29 years	1029 (64.2)	11.3
	30+ year	488 (30.4)	18.0
Age at first marriage			
	Under 20 years	332 (20.7)	15.4
	20–29 years	1181 (73.7)	12.1
	30+ years	90 (5.6)	21.1
If ever married–you choose your husband			
	No	973 (60.7)	13.2
	Yes	630 (39.3)	13.5
If ever married–if you did not choose your husband, you gave your consent to the choice			
	No	30 (1.9)	6.7
	Yes	1572 (98.1)	13.4
	Not Stated	1 (0.1)	0.0
Ethnic group			
	Memon	209 (13)	12.4
	Sindhi	365 (22.8)	14.2
	Katchi	54 (3.4)	14.8
	Gujrati	56 (3.5)	7.1
	Punjabi	123 (7.7)	17.1
	Balochi	65 (4.1)	12.3
	Pathan	76 (4.7)	10.5
	Urdu-Mahajir	502 (31.3)	13.1
	Other	153 (9.5)	13.1
Location			
	Karimabad	448 (27.9)	9.4
	Garden	334 (20.8)	15.6
	Kharadar	348 (21.7)	15.8
	Hyderabad	473 (29.5)	13.5
Education completed			
	Primary or lower	156 (9.7)	19.2
	Secondary/high school	286 (17.8)	12.9
	College/university	621 (38.7)	11.6
	Post graduate degree	540 (33.7)	13.7
Income			
	Low-income	273 (17)	15.4
	Middle-income	465 (29)	12.3
	High-income	791 (49.3)	13.0
	Not stated	74 (4.6)	14.9
Occurrence of terrible events			
	No	1297 (80.9)	14.1
	Yes	306 (19.1)	9.8
Previous preterm birth			
	Primiparous	670 (41.8)	11.2
	No	860 (53.6)	14.0
	Yes	73 (4.6)	24.7
Sex of the child^a^			
	Boy	855 (53.3)	13.6
	Girl	744 (46.4)	12.9
Family history of preterm birth			
	No preterm birth in family	1400 (87.3)	13.9
	Preterm birth in husband’s family	70 (4.4)	10.0
	Preterm birth in mother’s family	133 (8.3)	9.0
Planned Pregnancy			
	No	375 (23.4)	13.1
	Yes	1228 (76.6)	13.4
Mother’s employment			
	Homemaker	1428 (89.1)	13.6
	Non-government employee	99 (6.2)	11.1
	Other	76 (4.7)	10.5
Father’s employment			
	Government employee	205 (12.8)	12.2
	Non-government employee	796 (49.7)	12.7
	Self-employed	560 (34.9)	14.1
	Other	42 (2.6)	19.0
Food insecurity (i.e., missed food for ≥ 8 hours)			
	No	1379 (86)	13.6
	Ramadan, fasting	118 (7.4)	11.0
	Various reasons	106 (6.6)	12.3
Socio-economic Status			
	Low	126 (7.9)	19.8
	Middle	862 (53.8)	13.5
	High	615 (38.4)	11.7
Drugs (before or during pregnancy)			
	No	1387 (86.5)	13.0
	Yes	216 (13.5)	14.8
Smoke (before or during pregnancy)			
	No	1591 (99.3)	13.2
	Yes	12 (0.7)	25.0
Substance use (lifetime)			
	No	1368 (85.3)	13.0
	Yes	235 (14.7)	14.9
Trauma			
	No	1218 (76)	13.4
	Yes	385 (24)	13.0
Social support from family			
	No	78 (4.9)	12.8
	Yes	1525 (95.1)	13.3
Social support from friends			
	No	242 (15.1)	12.8
	Yes	1360 (84.9)	13.4
Social support from other source			
	No	9 (0.6)	11.1
	Yes	1593 (99.4)	13.3
Sexual abuse			
	No	1532 (95.6)	13.4
	Yes	71 (4.4)	11.3
Emotional abuse			
	No	1518 (94.7)	13.2
	Yes	85 (5.3)	14.1
Physical abuse			
	No	1390 (86.7)	13.1
	Yes	213 (13.3)	14.6
Sexual/emotional abuse			
	No	1467 (91.5)	13.3
	Yes	136 (8.5)	13.2
Pregnancy-related anxiety			
	No	1486 (92.7)	13.5
	Yes	117 (7.3%)	10.3
Antenatal state anxiety			
	No	1559 (97.3)	13.2
	Yes	44 (2.7)	15.9
Antenatal depression			
	No	1397 (87.1)	13.7
	Yes	206 (12.9)	10.7
Chronic stress (PSS ≥ 20)			
	No	961 (60)	15.0
	Yes	642 (40)	13.3
Co-morbidities			
	None	1302 (81.2)	13.7
	One (any one psychosocial distress condition)	242 (15.1)	12.4
	Two (any two psychosocial distress conditions)	52 (3.2)	7.7
	Three (all three psychosocial distress conditions)	7 (0.4)	14.3

Note. PSS = Perceived Stress Scale.

^a^ Data available for 1599 (99.8%).

A total of 213 live birth were preterm (prevalence rate = 13.3%, 95% CI: 11.7–15.0%). In total, 178 (83.6%) of the 213 women with preterm births had no antenatal psychosocial distress conditions, while 18 of the remaining 35 women with one or more antenatal psychosocial distress conditions experienced only symptoms of depression and no state anxiety or pregnancy-related anxiety. Potential confounders and effect modifiers of preterm birth and psychosocial distress conditions are shared in S1 Table.

### Model for preterm birth given individual antenatal psychosocial distress condition

Individually, neither depression nor state anxiety were significant predictors of preterm birth. The adjusted OR (aOR) remained unchanged and non-significant when confounders including age, location, occurrence of terrible events in the neighbourhood were retained in each of the models, and none of the effect modifiers were significant after adjusting for significant confounders. The crude effects of pregnancy-related anxiety were not significant and remained non-significant after adjusting for potential confounders. When potential effect modifiers (income, food availability, social support from family, current planned pregnancy) and their interactions were considered, planned pregnancy emerged as the only significant effect modifier (*p* = 0.021) of the effect of pregnancy-related anxiety on preterm birth. The odds of women who experienced pregnancy-related anxiety, and who had not planned for pregnancy, experiencing preterm birth was over 4 times (OR = 4.61, 95% CI: 1.25–16.92) greater than the corresponding odds for women who did not experience pregnancy-related anxiety and who had planned for pregnancy. The effect of having previous preterm birth was significant (*p* = 0.004), but the interaction term, a measure of the extent of effect modification, was not significant (*p* > 0.1) and hence dropped from the model using variable selection (See Table 2). Hence, pregnancy-related anxiety is a potential predictor of preterm birth among women with unplanned pregnancy after adjusting for age, occurrence of terrible events in the neighbourhood, and history of preterm birth.

**Table 2:** Model for preterm birth given pregnancy-related anxiety.

Outcome: Preterm birth Variable and role			Crude estimate		Adjusting for confounding		Adjusting for confounding and effect modifications	
			*p* Value	OR (95% CI)	*p* Value	OR (95% CI)	*p* Value	OR (95% CI)
**Predictor**								
	Pregnancy-related anxiety (ref = No)		0.318	0.73 (0.39–1.35)	0.382	0.76 (0.4–1.42)	0.050	0.39 (0.15–1.00)
**Confounders**								
	Age at enrolment (Years)				0.001	1.05 (1.02–1.09)	0.003	1.05 (1.02–1.09)
	Location (ref = Karimabad)				0.024		0.026	
	Garden				0.012	1.76 (1.13–2.74)	0.013	1.77 (1.13–2.8)
	Kharadar				0.009	1.78 (1.15–2.76)	0.007	1.87 (1.19–2.93)
	Hyderabad				0.011	1.73 (1.13–2.65)	0.0178	1.68 (1.1–2.59)
	Occurrence of terrible events (ref = No)				0.048	0.63 (0.40–0.99)	0.028	0.6 (0.38–0.95)
	Previous preterm birth (ref = No)						0.009	
	No: Primiparous						0.846	1.03 (0.74–1.45)
	Yes						0.004	2.51 (1.35–4.65)
**Effect modifiers**								
		Current pregnancy planned (ref = Yes)					0.685	0.92 (0.63–1.36)
		Pregnancy not planned and pregnancy-related anxiety					0.021	4.61 (1.25–16.92)

Note. OR–odds ratio, CI–confidence intervals, ref–reference

### Model for preterm birth given multiple antenatal psychosocial distress conditions

We developed the model for preterm birth with all three antenatal psychosocial distress conditions as predictors and adjusted for (a) all their confounders entering age, location, and occurrence of terrible of events, and (b) effect modifiers entering experience of previous preterm birth and planned pregnancy which were effect modifiers of pregnancy-related anxiety (see Table 2). In this analysis, we also investigated if perceived stress, a representative measure of chronic stress, was a potential effect modifier for any of the three antenatal psychosocial distress conditions. As was the case for the individual models, none of the main or interactive effects of the three antenatal psychosocial distress conditions showed significance as predictors of preterm birth. These non-significant interactive effects were excluded, and perceived stress evaluated to determine if it confounded or modified the potential effects of any of the antenatal psychosocial distress conditions. Age (*p* = 0.002), location (*p* = 0.041), occurrence of terrible events in the neighborhood or community (*p* = 0.028), previous preterm birth (*p* = 0.008), chronic stress (*p* = 0.015), and the interaction between pregnancy-related anxiety and unplanned pregnancy (*p* = 0.029) emerged as the significant effects in the model. None of the interactive effects between chronic stress and individual antenatal psychosocial distress conditions were significant. The final model is shown in Table 3. Although the effects of depression were not significant, when depression or its interaction with chronic stress were dropped from the model, the effect of chronic stress became non-significant. Comparing the model with and without depression, the change in likelihood trended towards significance (*p* = 0.095). However, the AIC was unchanged at 1239 for both the sub model and the full model.

**Table 3:** Final model for preterm birth given antenatal psychosocial distress conditions at time 1.

Outcome: Preterm birth		*p* Value	OR (95% CI)
Variable and role			
**Predictors**			
	Pregnancy-related anxiety (ref = no)	0.255	1.69 (0.68–4.21)
	Depression (ref = no)	0.630	1.21 (0.55–2.66)
**Covariates**			
	Age at enrolment (Years)	0.002	1.05 (1.02–1.09)
	Location (ref = Karimabad)	0.041	
	Garden	0.007	1.89 (1.19–3.00)
	Kharadar	0.025	1.70 (1.07–2.70)
	Hyderabad	0.107	1.45 (0.92–2.27)
	Occurrence of terrible events (ref = no)	0.028	0.60 (0.38–0.95)
	Previous preterm birth (ref = no)	0.008	
	No: Primiparous	0.825	0.96 (0.68–1.35)
	Yes	0.003	2.47 (1.37–4.47)
**Effect modifiers**			
	Chronic stress (ref = no)	0.015	1.57 (1.09–2.25)
	Chronic stress and depression	0.103	0.44 (0.16–1.18)
	Planned pregnancy (ref = no)	0.659	1.09 (0.74–1.61)
	Planned pregnancy and pregnancy-related anxiety	0.029	0.23 (0.06–0.86)

Note. OR–odds ratio, CI–confidence interval, ref–reference

These analyses reveal that among the three antenatal psychosocial distress conditions, only pregnancy-related anxiety had some effect on preterm birth. The effect of pregnancy-related anxiety is significant in distinguishing effects of pregnancy-related anxiety among women who planned for pregnancy compared to all other women. Hence, pre-planned pregnancy reduced the effect of pregnancy-related anxiety on preterm birth by up to 77% (OR = 0.234), although the true reduction could range from as little as 6% to as high 86%.

The odds of having preterm birth among women who reported pregnancy-related anxiety, but had planned their pregnancy, was 0.234 (95% CI: 0.06–0.86, *p* = 0.029) compared to the odds of preterm birth among women who did not report pregnancy-related anxiety and had not planned for pregnancy. The odds of preterm birth among all other women (anxious and had not planned for pregnancy (OR = 1.695, *p* = 0.255); and anxious and had planned for pregnancy (OR = 1.091, *p* = 0.659)) were not significantly different from the odds of preterm birth among women who were not anxious and had not planned for pregnancy.

### Composite measure of antenatal psychosocial distress conditions a predictor of preterm birth

Analysis of the composite measure obtained by adding the 3 indicators revealed that 1302 of women (81.2%) had a score of 0, and only 59 (3.7%) had a score of 2 or 3. Thus, the overall prevalence of antenatal psychosocial distress was 18.8%, while prevalence of comorbidity of the 3 dimensions was only 3.7%. When this binary variable was used as the sole predictor, the crude odds-ratio was not significant (OR = 0.831, p = 0.347). Findings (Table 4) indicate that the binary version of the composite measure of perinatal anxiety and depression was not predictive of preterm birth. Even after adjusting for potential confounders and effect modifiers, the adjusted odds-ratio remained non-significant (aOR = 1.54, *p* = 0.381) and while interactions with potential effect modifiers of planned pregnancy (*p* 0.350) and previous preterm birth (p = 0.868) were also not significant. The interaction with chronic stress (*p* = 0.098) trended towards significance. Hence, aggregate antenatal psychosocial distress did not improve model performance over pregnancy-related anxiety. The AIC for this new model was 1244 compared to 1239 obtained in our final model, indicating that it is indeed a poorer fit to the data.

**Table 4:** Model for preterm birth given aggregate antenatal psychosocial distress measures.

		*p* value	OR (95% CI)
**Predictor**			
	Aggregate APDM (ref = None)	0.381	1.54 (0.59–4.07)
**Confounders**			
	Age (Yrs)	0.002	1.05 (1.02–1.09
	Location (ref = Karimabad)	0.029	
	Garden	0.004	1.98 (1.24–3.14)
	Kharadar	0.027	1.69 (1.06–2.70)
	Hyderabad	0.091	1.47 (0.94–2.30)
	Terrible events (ref = No)	0.032	0.61 (0.38–0.96)
**Potential Effect modifiers**			
	Planned pregnancy (ref = No)	0.790	1.06 (0.70–1.61)
	Planned pregnancy by aggregate APDM	0.350	0.67 (0.28–1.56)
	Previous preterm birth baby (ref = No)	0.022	
	YES-Preterm baby	0.008	2.47 (1.26–4.85)
	Primiparous	0.736	0.94 (0.65–1.35)
	Previous preterm birth by aggregate APDM	0.868	
	Yes-Preterm baby by 1–3 APDM	0.724	0.78 (0.19–3.13)
	Primiparous by 1–3 APDM	0.752	1.15 (0.48–2.73)
	Chronic stress (ref = None)	0.013	1.60 (1.10–2.33)
	Chronic stress by 1–3 APDM	0.098	0.49 (0.21–1.14)

Note. OR–odds ratio, APDM–antenatal psychosocial distress measures, CI–confidence interval, ref—reference

## Discussion

Pregnancy-related anxiety was predictive of preterm birth among women with unplanned pregnancy after adjusting for the effects of age, occurrence of terrible events in the neighborhood, and history of preterm birth. Neither depression nor state anxiety were significant predictors of preterm birth after adjusting for confounders (age, location, and occurrence of terrible events in the neighborhood). Patterns of antenatal distress conditions (one or more psychosocial distress measure) did not improve model prediction of preterm birth. Our findings are contrary to studies from both LMIC and high-income countries, which have demonstrated an association with either depression, state anxiety, or comorbid psychosocial distress conditions during pregnancy and preterm birth [12, 39, 58].

When examining preterm birth from an evolutionary perspective, it is suggested that adverse maternal contextual factors (e.g., socio-economic status, chronic stress) may initiate adaptive responses that, in conjunction with psychosocial distress, may determine the trajectory of preterm birth [75]. The risk of preterm birth has been reported to increase when depression occurs in combination with other etiological contributing factors (e.g., parity, household socio-economic status) [76]. Our study identified four potential effect modifiers: income, food availability, social support from family, and current planned pregnancy. Pregnancy-related anxiety became an important predictor of preterm birth when interactive effects were considered with current pregnancy not being planned emerging as the only significant effect modifier (i.e., increasing odds over 4 times) of the effect of pregnancy-related anxiety on preterm birth. This finding emphasizes importance of women’s sexual and reproductive health care in the prevention of preterm birth.

In our sample, 23.4% of women had unplanned pregnancies, which is comparable to other Pakistani studies [47, 77] but higher than rates reported among women in six South Asian countries [78]. Further analysis of our data revealed an association between planned pregnancy and income (*p* = 0.003), parity (*p* < 0.001), age (*p* < 0.001) and years in marriage (*p* = <0.001). Curiously, women with low total household income were more likely to have planned pregnancy (81%) compared to women with high total household income (72.9%). Women who planned pregnancy were about 1 year older, but had been married for about 1 year less, compared to women who did not plan their pregnancy. Primiparous women were the most likely to have planned their pregnancy (84%) compared other women. Furthermore, the more the number of previous children, the less likely the woman would plan for pregnancy. Our findings illustrate the importance of considering the socio-cultural context of women when examining the relationship between antenatal psychosocial distress and preterm birth.

The nature of psychosocial distress and contextual factors in and of themselves does not lead to preterm birth directly; rather, these factors are mediated through biological pathways. These biological pathways may include multisystem dysregulations that initiate pathological processes of stress-related medical conditions during pregnancy (e.g., gestational hypertension and diabetes) that can induce preterm birth [52]. Only 3.7% of our sample self-reported one or more medical conditions during pregnancy with gestational diabetes being most common (n = 21 with 2 women experiencing preterm birth), followed by gestational hypertension (n = 15 with 4 experiencing preterm birth), and persistent vaginal bleeding (n = 11 with 4 experiencing preterm birth). Also, given the infrequent occurrence of preterm birth with each condition the variable was not included in the analyses. Women differ in how they respond to stressful situations and these individual differences in physiological stress reactivity may explain why not all women who experience stress will have a preterm birth [52].

Early life experiences and chronic stress can have long-term effects on the hypothalamic-pituitary-adrenal axis, such as altering basal cortisol levels, and impacting psychosocial and biological responses to new stressful stimuli later in life (i.e., pregnancy) [79, 80]. These allostatic systems may initiate four types of responses–repeated hits, lack of adaptation, prolonged response, and inadequate response–to adapt to stressors [81]. The brain is central in adapting, through neural circuitry plasticity, to increase stress-related pathways to disease (i.e., preterm birth) or resilience against pathways to preterm birth [81]. Our study did not consider protective or strength-oriented factors, such as resilience, an enduring dynamic process, which entails adapting mentally, behaviorally, and biologically to stress, adversity, and threats [82–84]. In our study, chronic stress (i.e., Perceived Stress Scale ≥ 20), which was experienced by 39% of the sample, demonstrated no significant interactive effects with individual antenatal psychosocial distress measured during pregnancy and preterm birth. We have also demonstrated no relationship between adverse life experiences and preterm birth in a diverse sample of 300 low-risk pregnant women recruited from the same sites [85]. Hence, it is plausible that women’s resilience was an important factor that buffered the effects of perinatal mental distress and preterm birth in our sample of Pakistani women.

Pregnancy is a dynamic state as there is a dampening of psychological and biological responses to psychosocial distress late in the second trimester [86–88]; thus, a more complex relationship may exist between antenatal psychosocial distress and preterm birth. Studies [86, 89, 90] have determined that patterns of psychosocial distress over the course of pregnancy, such as change in state anxiety and perceived stress [86], and magnitude of change [90], may be better predictors of preterm birth than single timepoint assessments during pregnancy. Our future work will examine these relationships.

### Limitations and strengths

Our findings and conclusions cannot necessarily be generalized to other LMIC, especially because socio-cultural context has been identified as an effect modifier and differs in other parts of the world. Our eligibility criteria excluded women experiencing unique bio-psycho-social dynamics (e.g., those who were victims of terrorism, achieved pregnancy with aid of artificial reproductive technologies) thus may have introduced selection bias. We did not consider protective or strength-oriented factors, such as resilience, which may explain the findings of our study. We have also not explored the adaptive responses of the fetus to the in-utero environment and the advantage to the fetus in continuing pregnancy to term, particularly since in LMIC preterm birth and its complications contribute to neonatal and childhood mortality [91]. Our previous work has demonstrated acceptable reliability of the overall score of the Edinburgh Perinatal Depression Scale and pregnancy-related anxiety used in this study [92]. Our study is unique in that it prospectively explored the nature and multiplicative effects (i.e., multicollinearity) of types of antenatal psychosocial distress on preterm birth in the same sample of pregnant women from Pakistan.

## Conclusions

Psychosocial distress during pregnancy was not associated with preterm birth; however, like studies in high-income countries, pregnancy-related anxiety became a strong predictor of preterm birth when considering interactive effects of whether the current pregnancy was planned. Women’s ability to make decisions regarding their reproductive health (i.e., pregnancy-related agency), contraceptive care, and ways in which women respond to or face antenatal psychosocial distress and stress, are important to integrate in future research examining the relationship between antenatal psychosocial distress among Pakistani pregnant women and preterm birth. Future research should examine the intersections between women’s empowerment viewed through a multi-dimensional construct, antenatal psychosocial distress, and preterm birth [49].

## Supporting information

S1 Table P values from chi-square tests of association to determine potential confounders and effect modifiers of individual antenatal psychosocial distress conditions. (DOCX)
