# Supplementary material for: Individual and collective contribution of antenatal psychosocial distress conditions and preterm birth in Pakistani women
Source: PLoS One. 2023 Mar 30;18(3):e0282582. doi: 10.1371/journal.pone.0282582 (PMC10062634; doi:10.1371/journal.pone.0282582)
Supplement: S1 Table — (DOCX) [file pone.0282582.s001.docx]

**S1 Table 1.** **P values from chi-square tests of association to determine potential confounders and effect modifiers of individual antenatal psychosocial distress conditions.**

|  |  |  | **Preterm Birth** | **Pregnancy- related anxiety** | **Antenatal state anxiety** | **Antenatal depression** |
| --- | --- | --- | --- | --- | --- | --- |
| **Role^a^** | **Potential confounder/effect modifier** | df | Yes = 213 No = 1390 | Yes = 117 No = 1486 | Yes = 44  No = 1559 | Yes = 206  No = 1397 |
| Co | Location | 3 | **0.024** | **<0.001** | **0.003** | **<0.001** |
| Co | Were any of these children born preterm | 1 | **0.004** | 0.309 | **0.060** | **0.020** |
| Co | Occurrence of terrible events in neighborhood or community | 1 | **0.046** | **<0.001** | **<0.001** | **0.015** |
| Em | Socio-Economic Status | 2 | **0.049** | 0.580 | 0.265 | 0.222 |
| Em | Age^b^ at enrolment | 1 | **0.004** | 0.259 | **0.050** | 0.490 |
| Em | Was the current pregnancy planned | 1 | 0.822 | **0.032** | **0.005** | **0.006** |
| Em | No social support from family | 1 | 0.899 | **0.045** | **<0.001** | **<0.001** |
| Em | Reason for missing food for 8 or more hours | 2 | 0.700 | **<0.001** | **<0.001** | **<0.001** |
| Em | Life-time interpersonal trauma | 1 | 0.842 | 0.515 | **0.003** | **<0.001** |
| Em | If ever married - you choose your husband | 1 | 0.806 | **0.037** | 0.265 | **0.009** |
| Em | Ethnic Group | 8 | 0.813 | 0.254 | 0.179 | **<0.001** |
| Em | No social support from friends | 1 | 0.809 | 0.919 | 0.136 | **<0.001** |
| Em | No other social support | 1 | 0.847 | 0.641 | **<0.001** | 0.884 |
| Em | Household income level | 3 | 0.642 | 0.321 | **0.005** | 0.120 |
| Em | Father’s employment | 3 | 0.573 | **0.005** | 0.353 | 0.313 |
| Em | Mother’s employment | 2 | 0.601 | **0.011** | 0.665 | 0.345 |
| Em | Lifetime mental illness | 1 | 0.720 | **0.014** | **<0.001** | **<0.001** |
| Em | Abuse sexual | 1 | 0.608 | **0.025** | 0.435 | **<0.001** |
| Em | Abuse emotional | 1 | 0.817 | 0.442 | **0.001** | **<0.001** |
| Em | Abuse physical | 1 | 0.559 | 0.898 | 0.332 | **<0.001** |
| Em | Abuse any | 1 | 0.985 | 0.036 | **0.004** | **<0.001** |
| Ne | History of preterm birth | 2 | 0.207 | 0.069 | 0.078 | **0.024** |
| Ne | Highest level of education completed | 3 | 0.092 | 0.907 | 0.157 | 0.405 |
| Ne | Drugs before or during pregnancy | 1 | 0.477 | 0.335 | 0.609 | 0.801 |
| Ne | Smoked before or during pregnancy | 1 | 0.230 | 0.869 | 0.564 | 0.680 |
| Ne | Lifetime substance use | 1 | 0.432 | 0.268 | 0.784 | 0.564 |
| Ne | Number of terrible Events in neighborhood or community | 2 | 0.786 | 0.532 | 0.897 | 0.604 |
| Ne | Baby mother Occupation | 2 | 0.722 | 0.078 | 0.863 | 0.199 |
| Ne | Age at first marriage | 1 | 0.784 | 0.816 | 0.906 | 0.589 |
| Ne | If every you married – you gave your consent of choice^*^ | 1 | 0.292 | 0.569 | 0.002 | 0.317 |
| Ne | Sex of baby^**^ | 1 | 0.696 | 0.026 | 0.439 | 0.748 |
| Ne | Baby’s mother’s occupation | 2 | 0.722 | 0.093 | 0.847 | 0.192 |

Note. Co – potential confounder, Em – potential effect modifier, Ne – no potential effect

^a^ Roles were evaluated in multiple logistic regression models using forward variable addition to include only significant factors and significant interactions. *P* value was obtained from simple logistic regression.

^b^ Age is in years.

* Only 4 women out of 30 women did not give consent experienced state anxiety. This is too few to analyze in a multi-variable analysis with 3 or more variables (preterm birth, anxiety, ever married).

** Sex of baby may not meet condition for confounding as it might be interpreted as being the pathway between anxiety and preterm birth.
